# Environmental chemicals and DNA methylation in adults: a systematic review of the epidemiologic evidence

**DOI:** 10.1186/s13148-015-0055-7

**Published:** 2015-04-29

**Authors:** Adrian Ruiz-Hernandez, Chin-Chi Kuo, Pilar Rentero-Garrido, Wan-Yee Tang, Josep Redon, Jose M Ordovas, Ana Navas-Acien, Maria Tellez-Plaza

**Affiliations:** Department of Internal Medicine, Hospital Clínico de Valencia, Avenida Blasco Ibañez, 17, 46010 Valencia, Spain; Area of Cardiometabolic and Renal Risk, Institute for Biomedical Research Hospital Clinic de Valencia INCLIVA, Av. Menendez Pelayo 4, Accesorio, 46010 Valencia, Spain; Department of Environmental Health Sciences, Johns Hopkins University Bloomberg School of Public Health, 615 North Wolfe Street, Baltimore, MD 21205 USA; Department of Epidemiology, Johns Hopkins Bloomberg School of Public Health, 615 North Wolfe Street, Baltimore, MD 21205 USA; Department of Internal Medicine, Kidney Institute and Division of Nephrology, China Medical University Hospital and College of Medicine, China Medical University, 2 Yude Road, Taichung, 40447 Taiwan; Genotyping and Genetic Diagnosis Unit, Institute for Biomedical Research INCLIVA, Av. Menendez Pelayo, 4 Accesorio, 46010 Valencia, Spain; CIBER Physiopathology of Obesity and Nutrition (CIBEROBN), Institute of Health Carlos III, Minister of Health, Madrid, Spain; Nutrition and Genomics Laboratory, Jean Mayer US Department of Agriculture Human Nutrition Research Center on Aging at Tufts University, 711 Washington St, Boston, MA 02111-1524 USA; Instituto Madrileño de Estudios Avanzados en Alimentación, Ctra. de Cantoblanco 8, 28049 Madrid, Spain; Welch Center for Prevention, Epidemiology and Clinical Research, Johns Hopkins Medical Institutions, 2024 E. Monument Street, Baltimore, 21205 MD USA

**Keywords:** Systematic review, DNA methylation, Environmental chemicals, Cadmium, Lead, Mercury, Metals, Persistent organic pollutants, Bisphenol A, Polycyclic aromatic hydrocarbons

## Abstract

Current evidence supports the notion that environmental exposures are associated with DNA-methylation and expression changes that can impact human health. Our objective was to conduct a systematic review of epidemiologic studies evaluating the association between environmental chemicals with DNA methylation levels in adults. After excluding arsenic, recently evaluated in a systematic review, we identified a total of 17 articles (6 on cadmium, 4 on lead, 2 on mercury, 1 on nickel, 1 on antimony, 1 on tungsten, 5 on persistent organic pollutants and perfluorinated compounds, 1 on bisphenol A, and 3 on polycyclic aromatic hydrocarbons). The selected articles reported quantitative methods to determine DNA methylation including immunocolorimetric assays for total content of genomic DNA methylation, and microarray technologies, methylation-specific quantitative PCR, Luminometric Methylation Assay (LUMA), and bisulfite pyrosequencing for DNA methylation content of genomic sites such as gene promoters, LINE-1, Alu elements, and others. Considering consistency, temporality, strength, dose-response relationship, and biological plausibility, we concluded that the current evidence is not sufficient to provide inference because differences across studies and limited samples sizes make it difficult to compare across studies and to evaluate sources of heterogeneity. Important questions for future research include the need for larger and longitudinal studies, the validation of findings, and the systematic evaluation of the dose-response relationships. Future studies should also consider the evaluation of epigenetic marks recently in the research spotlight such as DNA hydroxymethylation and the role of underlying genetic variants.

**Electronic supplementary material:**

The online version of this article (doi:10.1186/s13148-015-0055-7) contains supplementary material, which is available to authorized users.

## Review

### Introduction

**Figure 1:**
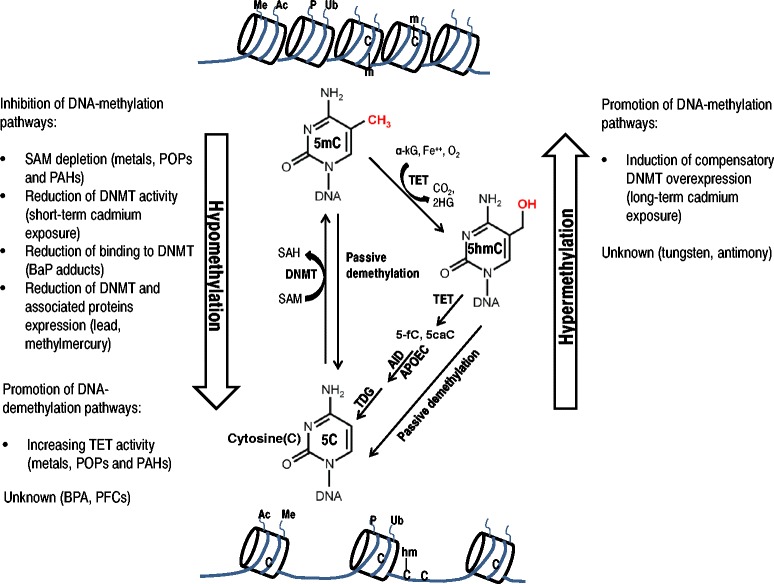
**Overview of possible mechanisms of action for environmental chemicals on DNA methylation based on reviews of experimental studies** **[** 2 **,** 3 **,** 5 **,** 135 **,** 136 **].** Metals, POPs, and PAH increase reactive oxygen species (ROS) formation. Under chronic consumption of glutathione (GSH) for conjugation with ROS, chemicals, and their metabolites, homocysteine is employed into GSH rather than methionine synthesis pathways, leading to a reduced synthesis of S-adenosylmethionine (SAM, a substrate for DNA methyltransferases (DNMT) which catalyzes the addition of the methyl group onto the 5-carbon cytosine (5C) to become 5-methylcytosine (5mC)). SAM depletion, thus, potentially inhibits DNA methylation and results in subsequent DNA hypomethylation [2]. Exposures to specific environmental chemicals such as short-term cadmium, PAH, lead, and mercury exposures can directly reduce the enzymatic activity and concentrations of DNMT [136]. In addition, oxidative stress is proposed to stimulate the alpha-ketoglutarate (α-KG) production from isocitrate. α-KG activates ten-eleven translocation (TET) proteins that catalyze the oxidation of 5mC to 5-hydroxymethylcytosine (5hmC), 5-formlycytosine (5fC), and 5-carboxycytosine (5caC) in the presence of cofactors, iron and oxygen. 5hmC, 5fC, and 5caC could act as an intermediate in both passive and active DNA demethylation pathways [3,135] involving DNA repair enzymes like AID, APOEC, and TDG. Overall, it facilitates DNA hypomethylation. Conversely, it has been suggested that long-term cadmium exposure induces compensatory DNMT overexpression [4] that could lead to increased DNA methylation. On the other hand, environmental chemicals can modulate the enzymes involved in covalent modifications (acetylation (Ac), methylation (Me)), phosphorylation (P) and ubiquitination (Ub)) at the histone tails that can interact with the DNA methylation or demethylation machinery. Lead has been related with transcription-active histone modifications (associated to DNA hypomethylation), while methylmercury and nickel have been related with transcription-repressive histone modifications (associated to DNA hypermethylation) [5,136]. Finally, while other environmental toxicants have been related to DNA hypomethylation (BPA, PFCs) and hypermethylation (tungsten, antimony) in epidemiologic studies, their mechanism of action in epigenetic regulation of gene transcription is unknown.

Beyond lifestyle determinants, the role of environmental chemicals as determinants of DNA methylation has gained considerable attention. Changes in DNA methylation add biological plausibility to the increasingly recognized contribution of environmental chemicals to disease burden [1] as DNA methylation is involved in regulating many cellular processes, including X-chromosome inactivation, genomic imprinting, chromosome stability, and gene transcription. Environmental chemicals can interfere with the one-carbon and citric acid metabolism pathways, resulting in anomalous DNA-methylation status throughout the genome [2,3]. Environmental chemicals can also directly interact with enzymes involved not only in one-carbon metabolism and citric acid metabolism pathways but also in histone modifications [4-6]. A summary of suggested mechanisms of action of environmental chemicals on DNA methylation machinery is shown in Figure 1. In turn, these epigenetic mechanisms may modify potential toxicity pathways specific to the environmental chemicals in the organism.

Environmental chemicals have been linked to aberrant changes in epigenetic pathways both in experimental and epidemiological studies. In animal studies, maternal diet during pregnancy was associated with the pattern of DNA methylation of specific genes, which resulted in permanent phenotypic changes including body weight and blood pressure levels [7,8]. In humans, populations exposed to famine during the prenatal period showed increased prevalence of cardiometabolic factors and ischemic heart disease mortality [9], with evidence supporting a mediating role of epigenetic mechanisms in disease pathogenesis [10]. Deleterious effects of epigenetic changes are not restricted to the prenatal period. Monozygotic twins experienced an epigenetic drift in relation to one another with advancing age, time shared together, and behavioral factors such as smoking [11]. There is, however, a need to undertake a systematic appraisal of the epidemiologic evidence evaluating the potential role of environmental chemicals as determinants of DNA methylation in adults.

Our objective was to conduct a systematic review and synthesis of results from epidemiologic studies evaluating the association of environmental chemicals including cadmium, lead, mercury, nickel, persistent organic pollutants (POPs), bisphenol A (BPA), polycyclic aromatic hydrocarbons (PAHs), and phthalates, with DNA methylation levels in adults. We did not include arsenic studies in our search because there is a recently published systematic review published by Bailey *et al*. [12]. Other environmental exposures, which have been related to DNA methylation, such as exposure to tobacco smoke [13-17] and air pollution [18], are out of the focus of the present review, as tobacco smoke and air pollution are mixtures of different types of chemicals rather than individual groups of compounds.

### Methods

#### Search strategy, study selection, and data abstraction

**Figure 2:**
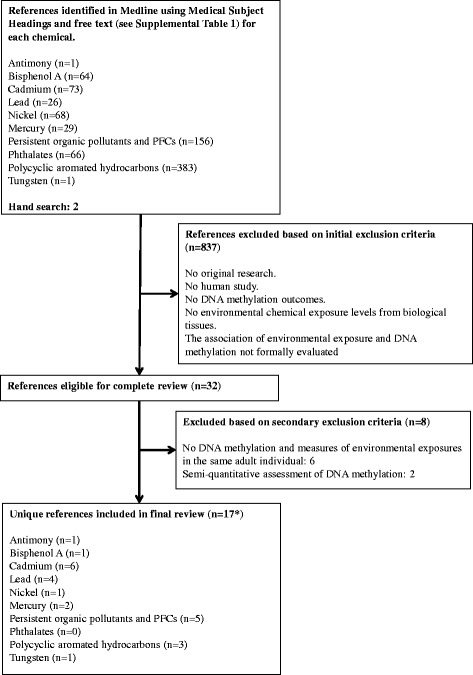
**Flow diagram of the study selection process.** Summary of inclusion and exclusion criteria used in this systematic review of studies investigating the association between environmental chemicals and DNA methylation levels, 10 April 2014. *17 references include the following studies with multiple environmental toxicants evaluated in unique study populations: Hanna *et al*. (2012) [29] examined in SMART population urine cadmium, blood lead and mercury, and serum BPA. Tajuddin *et al*. (2013) [30] examined in EPICURO population toenail cadmium, nickel, and lead. Tellez-Plaza *et al*. (2014) [19] examined in the SHS populations urine tungsten, antimony, and cadmium. Abbreviations: BPA, bisphenol A; PCF, perfluorinated compounds.

We searched PubMed for relevant studies published through 10 April 2014 using the search strategy described in Additional file 1: Table S1 (Supplemental Material). The search strategy retrieved a total of 867 citations (including duplicates). We included all articles assessing environmental chemical exposures using biomarkers. The search had no language restrictions. We also included two relevant studies published after 10 April 2014 and identified by hand search [19,20]. Two investigators (A.R.H. and C.C.K.) independently reviewed each of all the abstracts and selected 32 papers applying the following study exclusion criteria (Figure 2): a) no original research (that is, reviews, editorials, non-research letters); b) no human study; c) no DNA methylation outcomes; d) no environmental chemical exposure levels measured in biological tissues (for example, environmental measures such as water or air, or distance from a source). In this systematic review, the focus was on the role of environmental chemicals exposure in DNA methylation changes in adults. Therefore, as a second layer of exclusion, we additionally excluded one study focusing on prepubescent girls [21], and five studies that focused on the association of maternal exposure biomarkers and DNA methylation in cord blood or the offspring and did not provide corresponding measures of DNA methylation in the mothers [22-26]. We additionally, excluded two studies with semi-quantitative assessment of DNA methylation [27,28] as the comparison of results with quantitative DNA methylation assessment methods is unclear. Any discrepancies were resolved by consensus, and if necessary, a third reviewer was involved. A native speaker reviewed the full text of any non-English article that could not be included or excluded based on the initial abstract review. We included in the final review 17 papers, some of them measuring multiple environmental toxicants evaluated in unique study populations [19,29,30] (Figure 2). Our review identified no publications investigating the association between phthalates and DNA methylation. After retrieval of articles from the search, the reference lists of selected articles were checked for other potentially relevant articles, identifying no additional studies. We collected the following data for each study: first author, year of publication, study design, size and population characteristics, exposure assessment and categories for comparison, DNA methylation assessment and endpoint definition, measures of association and 95% confidence interval (CI) or *P* values, and statistical methods including DNA methylation raw data processing methods. For studies modeling exposures both as continuous and as categorical, we reported continuous measures of association due to space constraints in the tables. However, we evaluated flexible dose-response relationships when reported. For polychlorinated biphenyls (PCBs), when multiple congeners were reported, we selected the congener with the weakest, highest, and median association. We also reported all the statistically significant POPs.

To assess study quality, we adapted the criteria used by Longnecker *et al*. for observational studies (Supplemental Material, Additional file 2: Table S2) [31]. We followed the criteria proposed by the 2004 US Surgeon General Report on the health consequences of smoking [32], which include the evaluation of consistency, temporality, strength, dose-response relationship, and biological plausibility including confounding. As a result, the evidence for each environmental chemical and DNA methylation was classified into four groups as modified from the Surgeon General Report [32]: sufficient evidence, suggestive but not sufficient evidence, insufficient evidence to infer a relationship, and suggestive of no relationship. We organized the presentation of the results by environmental chemical.

#### Current perspectives and results

##### Cadmium and DNA methylation

Cadmium exposure from tobacco smoke, air pollution, occupation, and diet (leafy and root vegetables, grains, and offal) is widespread in general populations [33]. In the US, cadmium exposure has substantially decreased during the last decades, in part related to reductions in smoking [34]. Cadmium exposure, however, remains an important concern, because even at the currently reduced levels of exposure, cadmium has been related to cardiovascular, bone, and kidney disease in studies of the US National Health and Nutrition Examination Survey (NHANES) 1999 to 2008 data [35-41]. In epidemiologic studies, cadmium concentrations in blood and urine are established biomarkers of cadmium exposure and internal dose [33,42]. Both biomarkers can reflect cumulative exposure, although blood cadmium also reflects short-term fluctuations in exposure [33,42]. Experimental *ex vivo* evidence showed that cadmium was an effective, noncompetitive inhibitor of *M.SssI* DNA-methyltransferase (DNMT) (a bacterial DNMT that recognizes the same sequence as mammalian’s DNMTs) [4]. In rat liver cells, short-term cadmium exposure induced DNA global hypomethylation [4]. Prolonged exposure, however, resulted in global DNA hypermethylation [4,43-45]. In general, most *in vitro* and *in vivo* studies showed increased gene-specific DNA methylation after exposure to cadmium [46-52].

We identified six publications investigating the association between cadmium and DNA methylation (Table 1). These studies were conducted in the US [19,29,53], Argentina [54], Spain [30], and China [55]. Cadmium exposure was measured in urine only [19,29], blood only [53], both in urine and blood [54,55], and in toenail [30]. Global DNA methylation was assessed by pyrosequencing of LINE-1 elements (a surrogate marker of global DNA methylation) in three studies [29,30,54] and by an ELISA-like method (measurement of percent 5-methylcytocine [5-mC] in DNA sample) in one study [19]. CpG site-specific DNA methylation was measured in candidate genes by pyrosequencing in one study [55] and in an exploratory genome-wide manner using microarray technologies in two studies [53,54].

**Table 1:** **Studies of cadmium exposure biomarkers and DNA methylation outcomes (6 studies available)**

**First author, year**	**Design**	**Population**	**Size**	**Men (%)** ^**a**^	**Age Range (yr)** ^**a**^	**Exposure assessment**	**Exposure categories**	**DNA methylation Assessment**	**DNA methylation endpoint**	**Association**	**95% Confidence Interval or p-value**	**Adjustment Factors**
Hanna, 2012 [29]	CS	U.S. (Study of Metals and Assisted Reproductive Technologies [SMART])	42	0	Mean 36 (28 to 44)	Urine by DRC- ICPMS	Above and below the median	Whole blood	1,505 CpG sites percent methylation			Normalization. QC reported. BEE NR. CH partially addressed. Data unadjusted. MCC NR.
						Median = 0.38 μg/L		Site specific Illumina GoldenGate and bisulfite pyrosequencing of significant regions^b^		A trend towards hypermethylation if difference score > |30| (p < 0.05)No significant region^b^.		
								Global by bisulfite pyrosequencing of LINE-1		Approximately 0.2 % increase in median DNAm	p = 0.39	
Hossain, 2012 [54]	CS	Andean plateau, Northern Argentina	202	0	Median 34 (18-64)		Per log-unit increase	Whole blood	Average percent methylation	Difference		QC reported. CH not addressed. Only 4 participants were smokers. Regression models adjusted for age, coca chewing, and arsenic in urine. Cadmium concentrations corrected to the mean specific gravity of urine.
								Bisulfite pyrosequencing				
						Blood by DRC-ICPMS (Median = 0.36 μg/L)		Site specific				
								MLH1		0.19	−0.53, 0.91	
								CDKN2A		0.24	−0.29, 0.77	
								Global LINE-1		0.45	−0.23, 1.12	
						Urine by DRC-ICPMS (Median = 0.23 μg/L)		Site specific				
								MLH1		−0.073	−0.50, 0.36	
								CDKN2A		−0.11	−0.42, 0.21	
								Global LINE-1		−0.42	−0.82, –0.025	
Zhang, 2013 [55]	CS	Southern China	81	39.5	53.9 (IQR 48.0–59.0)	Graphite Furnace-AAS	Per log-unit increase	Whole blood				QC reported. CH not addressed. Regression models adjusted for age, sex, BMI, smoking, alcohol drinking, albumin, B2M, eGFR, N-acetyl-b-d glucosaminidase (NAG).
								Site specific by bisulfite pyrosequencing in:	Average percent methylation	Difference		
						Blood (Median = 2.62 μg/L)		RASAL1		0.49	0.21, 0.77	
								KLOTHO		1.18	0.54, 1.83	
						Urine by (Median = 5.20 μg/g creatinine)		RASAL1		0.88	0.57, 1.20	
								KLOTHO		1.55	0.75, 2.35	
Tajuddin, 2013 [30]	CS	Spain (EPICURO study)	659	89	66	Toenail by ICPMS (Median = 0.01 μg/g)	Per 1 μg/g increase	Blood granulocytes	Average percent methylation	Difference		QC reported. CH addressed. Adjusted for age, sex, study region, and smoking status
								Global by bisulfite pyrosequencing in LINE-1		0.1	−0.3, 0.6	
Sanders, 2014 [53]	Nested sub-CO	Durham county, US (CEHI study)	17	0	Maternal age: 28 (19–42)	Blood Median = 0.2 μg/L	Above and below the median	Blood leukocytes	Average percent methylation in 16 421 CpG islands	General pattern toward increased methylation with increased cadmium in 92 significant^c^ genes		Normalization. BEE NR. CH addressed. No adjustment conducted, but evaluation of participant characteristics by cadmium and DNAm levels, with no significant differences reported. FDR corrected q-value provided. SNP-related clustering of DNA methylation not evaluated.
								Site specific MBD2b/ MBD3L1 enrichment in Affymetrix Human Promoter 1.0R array				
										Fold-change of DNAm in top 5 significant sites:		
										TWSG1 = 1.79	0.0007	
										USP30 = 1.70	0.0023	
										FAM83H = 1.52	0.0052	
										PPP2R5B = 1.56	0.0060	
										PRKCG = 1.44	0.0068	
Tellez-Plaza, 2014 [19]	CS	13 American Indian communities, US (SHS)	48	31.3	55 ± 7.3	Urine by ICPMS Median = 0.87 μg/g	Above and below the median in 1989-1991	Global by ELISA-like commercial kit	Logit-transformed percent methylation relative to cytosine genomic content	Odds ratio		QC reported. Models adjusted for age, sex,smoking status, BMI and, in prospective analyses only, log-transformed total count of white blood cells and percent of neutrophils.
								Blood leukocytes in 1989–1991		1.75	0.96, 3.20	
	CO							Whole blood in 1997–1999		1.03	0.50, 2.11	

AAS: atomic absorption spectometry; BEE: batch effects evaluation; BMI: body mass index; CC: case-control; CH: Cell heterogeneity; CI: confidence interval; CO: cohort; CS: cross-sectional; DNAm, DNA methylation; FDR: false discovery rate; MCC: multiple comparison correction; NR: not reported; LOD: limit of detection; QC: quality control.

^a^Sociodemographic data available in the article, not necessarily in the subsample without missing values in DNA methylation or exposures.

^b^Significance was defined as a difference score > |13| (p < 0.05) and >10% absolute difference between the means for each group.

^c^Significance defined as a minimum absolute change of 30% comparing exposure groups and a p-value < 0.05.

In general, studies mostly showed a trend towards positive associations of cadmium exposure and DNA methylation. In a study population from Argentina (*N* = 200), however, blood cadmium was positively associated with DNA methylation in LINE-1 elements, but the association with urine cadmium was inverse [54]. Among five studies evaluating global or candidate gene methylation, three studies reported significant or marginally significant associations with cadmium biomarkers [19,54,55]. In US American Indians, the multi-adjusted odds ratio of percent 5-mC comparing participants with urine cadmium levels above and below 0.87 μg/g was 1.75 (95% CI 0.96, 3.20) [19]. In the Argentinean population, the difference in percent DNA methylation in LINE-1 elements per log-unit increase in urine cadmium was −0.42% (95% CI −0.82, −0.025) [54]. In a population from Southern China (*N* = 81) [55], the difference in average percent methylation in RASAL1 and KLOTHO genes per log-unit increase in urine cadmium was, respectively, 0.88% (95% CI 0.57, 1.20) and 1.55% (95% CI 0.75, 2.35). Both epigenome-wide association studies [29,53] evaluated general patterns in the association of DNA methylation in specific CpG sites and cadmium biomarkers in CpG sites with an effect size considered relevant, consistently finding a trend towards increased methylation with elevated cadmium exposure. In the Study of Metals and Assisted Reproductive Technologies (SMART) study, conducted in US women undergoing ovarian stimulation [29], no sites were considered significant. In the CEHI study, conducted in US mother-newborn pairs, percent increase in DNA methylation in the top five associated CpG sites ranged from 44% to 79% [53]. None of the genome-wide studies reported statistically significant regions after controlling for a false discovery rate, although the study sample sizes were relatively small [29,53]. Confounding by sex, age, and smoking status was generally addressed, with exceptions [29]. Only two studies [19,30] addressed the potential confounding effect of tissue cell heterogeneity.

##### Lead and DNA methylation

Lead in the environment has decreased over the last decades when regulations banning the use of lead in gasoline, paint, and solders were implemented [56,57]. The general population is exposed through ambient air, alcohol consumption, and tobacco smoke [58,59]. Patella and tibia lead are biomarkers of cumulative lead exposure and body burden, while blood lead is a biomarker of recent exposure including endogenous exposure from bone [60]. Patella lead is biologically more active than tibia lead [61], having a role in internal exposure dose from redistribution of accumulated lead in the body. Studies have shown associations between low-exposure to lead and increased risk of neurocognitive outcomes, high blood pressure, chronic kidney disease, hyperuricemia, gout, cardiovascular disease, cancer, and other health effects [60,62,63]. In *in vivo* and *in vitro* studies, lead exposure was associated with changes in DNA methylation and expression of specific genes [64-67], although experimental studies evaluating the molecular mechanisms of lead-induced changes in DNA methylation are needed.

We identified four publications investigating the association between lead and DNA methylation (Table 2). These studies were conducted in the US [29,68], China [69], and Spain [30]. Lead exposure was measured in blood [29,68,69], patella and tibia [68], or toenail [30]. Global DNA methylation was assessed by quantitative pyrosequencing of LINE-1 or Alu elements (Alu is another surrogate marker for global DNA methylation) in three studies [29,30,68] and by methylation specific real-time PCR in one study [69]. CpG site-specific DNA methylation was measured in an exploratory genome-wide manner using microarray technologies in one study [29], with validation of significant regions by quantitative pyrosequencing.

**Table 2:** **Studies of lead exposure biomarkers and DNA methylation outcomes (4 studies available)**

**First author, year**	**Design**	**Population**	**Size**	**Men (%)** ^**a**^	**Age Range (yr)** ^**a**^	**Exposure assessment**	**Exposure categories**	**DNA methylation Assessment**	**DNA methylation endpoint**	**Association**	**95% Confidence Interval or p-value**	**Data pre-processing and adjustment factors**
Hanna, 2012 [29]	CS	U.S. (Study of Metals and Assisted Reproductive Technologies [SMART])	24	0	Mean 36 (28 to 44)	Blood by DRC-inductively coupled plasma mass spectrometry	Above and below the median	Whole blood DNA	1,505 CpG sites percent metylation			Normalization. QC reported. BEE NR. CH partially addressed. Data unadjusted. MCC NR.
								Site specific Illumina GoldenGate and bisulfite pyrosequencing of significant regions^b^		A trend towards hypomethylation if difference score > |30| (*P* < 0.05)		
						Median = 0.73 μg/dL				COL1A2		
										38% decrease in mean DNA m r = - 0.45;	*P* = 0.004	
											*P* = 0.03	
								Global by bisulfite pyrosequencing of LINE-1		Approximately 0.1% increase in median DNAm	*P* = 0.76	
Tajuddin, 2013 [30]	CS	Spain (EPICURO study)	659	89	66	Toenail by ICPMS	Per 1 μg/g increase	Granulocyte DNA	Average % methylation	Difference		QC reported. CH addressed. Adjusted for age, sex, study region, and smoking status
						(Median = 0.40 μg/g)		Global by Quantitative pyrosequencing in LINE-1		−0.06	−0.1, 0.02	
Li, 2013 [69]	CS	Wuxi region, China	110	91	mean = 39.45 (range 20-55)	Blood by AAS		Peripheral leukocytes	Average % methylation		*P* <0.001	No QC reported. CH addressed and adjustments not reported.
							<100 μg/L	Global LINE-1 by methylation-specific real-time PCR		86.3%,		
							100-200 μg/L			78.6%		
							>200 μg/L			73.9%		
Wright, 2010 [68]	CO	US, Normative Aging Study	679	100	72.4			Buffy coat	Average % methylation	Difference		QC reported. Models adjusted for age, BMI, percent lymphocytes, education, smoking pack-years, and blood lead levels.
								Global by quantitative pyrosequencing				
						Tibia	Per IQR (15 μg/g) increase	LINE-1		−0.07	−0.29, 0.14	
								Alu		0.02	−0.10, 0.13	
						Patella	Per IQR (19 μg/g) increase	LINE-1		−0.25	−0.44, –0.05	
								Alu		−0.03	−0.14, 0.08	
						Blood	Per IQR (2 g/dL) increase	LINE-1		0.04	−0.10, 0.19	
								Alu		0.03	−0.05, 0.10	

AAS, atomic absorption spectrometry; BEE: batch effects evaluation; CH: Cell heterogeneity; DNAm, DNA methylation; IQR, interquartile range; LOD: limit of detection; MCC: multiple comparison correction; NR: not reported; QC: quality control.

^a^Sociodemographic data available in the article, not necessarily in the subsample without missing values in DNA methylation or exposures.

^b^Significance was defined as a difference score > |13| (p < 0.05) and >10% absolute difference between the means for each group.

In general, all the studies reported a trend towards inverse associations of lead exposure and global DNA methylation. Two studies reported statistically significant associations of DNA methylation with lead biomarkers [19,55]. In a Chinese population (*N* = 110), participants showed 86.3%, 78.6%, and 73.9% average LINE-1 methylation in blood lead groups including <100, 100 to 200, and >200 μg/L, respectively (*P* trend <0.001). In 678 men from the US Normative Aging Study, the absolute difference in average LINE-1 methylation percentage was −0.25% (95% CI −0.44, −0.05) per an interquartile range change (19 μg/g) in patella lead concentrations [68]. Blood and tibia lead biomarkers, however, did not show statistically significant associations with LINE-1 methylation in this study population, although the direction of the association was similar as compared to patella. The authors interpreted that the redistribution of accumulated lead from bone over time is associated with DNA methylation in circulating leukocytes. In the only epigenome-wide association study (*N* = 24) [53], a CpG site in the *COL1A2* gene showed decreased DNA methylation with elevated blood lead exposure under the established significance threshold. In pyrosequencing validation, this site showed a 38% decrease in average percent methylation (*P* value = 0.004) comparing individuals above and below 0.73 μg/dL of blood lead concentrations. Among CpG sites with an effect size considered relevant by the authors, a general trend towards hypomethylation with increasing blood lead levels was observed. There were not reported statistically significant regions after controlling for a false discovery rate [29]. Two (out of four) studies addressed potential confounding by sex, age, smoking status, and tissue cell heterogeneity in DNA methylation status [30,68]. While one of the studies was a cohort study with repeated measurements of lead biomarkers and DNA methylation [68], all the studies reported cross-sectional associations.

##### Mercury and other metals and DNA methylation

Mercury is a highly reactive metal with unknown physiological activity, which is persistent in the food chain [70]. While the main source of inorganic mercury is occupation (dentistry, mining, artisans manipulating mercury-containing materials) and dental amalgams, the general population is mainly exposed to organic mercury through consumption of fish (specially large predatory fish) and in a lesser degree shellfish and other marine animals [70]. Blood and hair mercury reflects exposure to methylmercury. Urine mercury, however, mainly reflects exposure to inorganic mercury [70]. Methylmercury is especially toxic for the neurologic system, especially during infancy [71]. Both methylmercury and inorganic mercury have immunotoxic effects, although the immunotoxicity is higher for inorganic mercury [71]. Other mercury-related health outcomes include cardiovascular disease, cancer, alterations of the reproductive system, and kidney disease [71-74]. There is evidence from experimental studies that mercury can change DNA methylation patterns. In rat embryonic neural stem cells and prenatally exposed adult rats, methylmercury reduced neural cell proliferation and was associated with global DNA hypomethylation [75]. In mouse stem cells, mercury exposure induced aberrant DNA methylation at specific gene loci [76]. The molecular mechanisms for potential epigenetic effects of mercury, however, are unknown.

Other nonessential metals are also of concern because they have been related to diverse health outcome in human studies. Tungsten has been related to cancer mortality [77], lung cancer, respiratory alterations, electrocardiograph abnormalities, and sudden death [78], and with prevalent cardiovascular disease and peripheral arterial disease [38,79]. Antimony was associated with peripheral arterial disease [38]. Nickel is an established carcinogen in occupational settings (respiratory cancers), especially insoluble nickel subsulfide and nickel oxide [80]. Other chronic health effects associated to nickel include rhinitis, sinusitis, nasal septum perforations, asthma, skin allergies, and reproductive effects [80]. However, experimental evidence indicating a potential role in altering DNA methylation for these metals is scarce, except for nickel. *In vitro* studies treatment with nickel resulted in both promoter hypermethylation and increased global DNA methylation [81,82]. Nickel may also influence DNA methylation by deregulating epigenetic enzymes involved in post-translational histone modifications [83,84].

For mercury, we identified two publications investigating the association between mercury and DNA methylation (Table 3). Both studies were conducted in the USA [29,85]. Mercury exposure was measured in blood [29] or urine and hair [85]. For other metals, we only identified one publication investigating the association of DNA methylation with toenail nickel in a population from Spain [30] and urine tungsten and antimony in US American Indians [19]. Among all the retrieved studies evaluating mercury and other metals, global DNA methylation was assessed by pyrosequencing of LINE-1 elements in three studies [29,30,85] and by and ELISA-like method in one study [19]. Site-specific DNA methylation was measured in candidate genes by pyrosequencing in one study [85] and in an exploratory genome-wide manner using microarray technologies in one study [29]. In 659 participants from the Spanish Bladder Cancer Study (EPICURO) [55], the difference in average percent methylation in LINE-1 elements per 1 μg/g increase in toenail nickel was 0.02% (95% CI 0.005, 0.03). In the only study reporting both cross-sectional and prospective associations, conducted in US American Indians [19], the odds ratio of global DNA methylation after 10 years of follow-up was 2.15 (95% CI 1.15, 4.01) comparing participants with baseline urine antimony levels above and below 0.27 μg/g. The cross-sectional association, however, was not statistically significant [19]. In one epigenome-wide association study in the SMART study population (*N* = 43) [53], only two CpG sites in the *GSTM1* gene showed increased DNA methylation with elevated blood mercury exposure under the established significance threshold of minimum absolute change of 10% and a *P* value <0.05. In pyrosequencing validation, CpG sites in this gene showed a 39% increase in average % methylation (*P* value = 0.04) comparing individuals above and below 2.88 μg/L of blood mercury concentrations. In this study, no statistically significant positions were reported after controlling for a false discovery rate [29]. The nickel, antimony, and tungsten [19,30], but not mercury [29,85], studies reported fully adjusted models including sex, age, and smoking status. For mercury, since the major source of exposure in humans is methylmercury from seafood consumption [86], adjustments for nutrients (for example, selenium, magnesium, n-3 fatty acids), lifestyle (seafood as a proxy for healthy diet), and other toxicants (POPs) in seafood should be considered. Only nickel, antimony, and tungsten studies [19,30] addressed the potential confounding effect of tissue cell heterogeneity.

**Table 3:** **Studies of mercury and other non-essential metals exposure biomarkers and DNA methylation outcomes (4 studies available)**

**First author, year**	**Design**	**Population**	**Size**	**Men (%)** ^**a**^	**Age Range (yr)** ^**a**^	**Exposure assessment**	**Exposure categories**	**DNA methylation Assessment**	**DNA methylation endpoint**	**Association**	**95% Confidence Interval or p-value**	**Data pre-processing and adjustment factors**
***Mercury***												
Hanna, 2012 [29]	CS	U.S. (Study of Metals and Assisted Reproductive Technologies [SMART])	43	0	Mean 36 (28 to 44)	Whole blood by DRC-ICPMS	Above and below the median	Whole blood DNA				Normalization. QC reported. BEE NR. CH partially addressed. Data unadjusted. MCC NR.
								Site specific Illumina GoldenGate and bisulfite pyrosequencing of significant regions^b^	1,505 CpG sites % methylation	A trend towards hypermethylation if difference score > |30| (p < 0.05)		
						Median = 2.88 μg/L						
										GSTM1 39% increase	p = 0.04	
										*r* pearson = 0.17	p = 0.27	
								Global by bisulfite pyrosequencing of LINE-1		~0.2% decrease in median DNAm	p = 0.42	
Goodrich, 2013 [85]	CS	US (Michigan Dental Association members)	131	49	55.8 ± 11.6	Total levels by direct Mercury Analyzer	Per log-unit increase	Buccal mucosa	Average % methylation	Difference		QC reported. Assessment of CH NR. Regression models adjusted for age and BMI.
								Quantitative pyrosequencing				
						Spot urine (Mean =0.7μg/L)		Site specific				
								DNMT1		−0.03	−0.32, 0.26	
								SEPW1		0.06	−0.12, 0.24	
								SEPP1		2.38	−1.23, 5.99	
								Global				
								LINE-1		0.37	−0.75, 1.49	
						Hair (Mean =0.37 μg/g)		Site specific				
								DNMT1		−0.13	−0.40, 0.14	
								SEPW1		−0.01	−0.19, 0.17	
								SEPP1		−2.02	−5.55, 1.51	
								Global				
								LINE-1		0.12	−0.96, 1.20	
***Other non-essential metals***												
Tajuddin, 2013 [30]	CS	Spain (EPICURO study)	659	89	66	Nickel	Per 1 μg/g increase	Granulocyte DNA	Average % methylation	Difference		QC reported. CH addressed. Adjusted for age, sex, study region, and smoking status
						Toenail by ICPMS		Global by Quantitative pyrosequencing in LINE-1				
						(Median =0.47 μg/g)				0.02	0.03, 0.005	
Tellez-Plaza, 2014 [19]	CS, CO	13 American Indian communities, US (SHS)	48	31.3	55 ± 7.3	Urine by ICPMS	Above and below the median at baseline	Global Methylamp Methylated DNA quantification kit (Epigentek)	Logit-transformed % methylation relative to cytosine genomic content	Odds ratio		QC reported. Models adjusted for age, sex, smoking status, BMI and, in prospective analyses only, log-transformed total count of white blood cells and percent of neutrophils.
						Antimony (Median = 0.27 μg/g)		Blood leukocytes in 1989–1991				
										1.24	0.71, 2.15	
						Tungsten (Median =0.13 μg/g)		Whole blood in 1997–1999		2.15	1.15, 4.01	
								Blood leukocytes in 1989–1991		1.46	0.85, 2.52	
								Whole blood in 1997–1999		0.93	0.46, 1.86	

BEE: batch effects evaluation; BMI: body mass index; CDT, Comparative Toxicogenomics Database; CC: case-control; CH: Cell heterogeneity; CI: confidence interval; CO: cohort; CS: cross-sectional; NR: not reported LOD: limit of detection; QC: quality control.

^a^Sociodemographic data available in the article, not necessarily in the subsample without missing values in DNA methylation or exposures.

^b^Significance was defined as a difference score > |13| (p < 0.05) and >10% absolute difference between the means for each group.

##### Persistent organic pollutants and other endocrine disruptors and DNA methylation

POPs are industrial chemicals that persist in the environment for decades even after production has been stopped [87]. The most well known are dioxins, PCBs, and polybrominated diphenyl ethers (PBDEs). Human exposure begins prenatally as many POPs can cross the placenta [88]. After birth, exposure occurs through breast milk [88] and also through inhalation (dust), ingestion (dairy and animal products), and skin contact [88,89]. POPs are lipophilic and accumulate in the adipose tissue. The potential effects of POPs include skin rashes to endocrine disruption, developmental delays, metabolic syndrome and diabetes, and cancer, depending on the type of compound and exposure [88].

Perfluorinated compounds (PFC) including perfluorooctanoic acid (PFOA) and perfluorooctanesulfonic acid (PFOS) are fluorocarbons with at least one additional atom or functional group and are included in the most recent list of POPs regulated by The Stockholm Convention [88]. For consistency with The Stockholm Convention and as previously done in other systematic reviews [90], we included PFCs in our search strategy for POPs. Drinking water is the primary route of PFCs exposure in some populations [91], but exposure sources are not well understood. While PFCs are persistent in the environment and in the body (half-life in humans is 3 to 5 years depending on the compound), they are not metabolized in humans and they are not lipophilic [91]. Animal data indicate that PFCs can cause several types of tumors and neonatal death and may have toxic effects on the immune, liver, and endocrine systems. Data on the human health effects include reported positive associations with cholesterol levels, hepatic enzymes, and adverse reproductive outcomes [91].

BPA is a compound with a shorter half-life compared to POPs, but it is frequently grouped together with POPs given its ubiquity and endocrine disruptor functions [88]. While humans are exposed through the placenta and ingestion (canned food), BPA is also present in dust and ambient air [88,92].

There are some studies evaluating the effect of POPs and other endocrine disruptors on DNA methylation in experimental settings. Exposure to dichlorodiphenyltrichloroethane (DDT) induced hypomethylation of CpG islands in *Sst, Gal, Arf1, Ttr, Msx1*, and *Grifin* genes in the hypothalamus of young male rats [93]. Rats treated *in utero* and postnatally with organochlorine pesticides and PCBs also showed decreased methylation of CpG sites in the promoter of the tumor suppressor gene *p16 (INK4a)* compared to controls [94]. Perfluorooctanoic acid induced gene promoter hypermethylation of *GSTP1* in human liver L02 cells [95]. Maternal BPA exposure disrupted genomic imprinting in the mouse embryos and placenta [96]. In rats, maternal exposure to BPA modified methylation of the metastable loci *Avy* and *CapbIAP* [97].

We identified four epidemiologic studies investigating the association between POPs [20,98-100], and one publication investigating PFCs [101] and BPA [29], respectively, with DNA methylation in adults (Table 4). These studies were conducted in the USA [29,101], South Korea [98], Sweden [99], Denmark [100], and Japan [20]. In studies assessing POPs, exposure was measured in plasma [100] or serum [20,98,99]. BPA was measured in serum [29] and PFCs were measured in blood [101]. Global DNA methylation was assessed by quantitative pyrosequencing of LINE-1 or Alu elements in four studies [29,98,100,101] and by Luminometric Methylation Assay (LUMA) in two studies [20,99]. CpG site-specific DNA methylation was measured in an exploratory genome-wide manner using microarray technologies in one study [29]. For most POPs, studies evaluating DNA methylation globally showed a trend towards hypomethylation with increasing levels of exposure [20,98,100]. In studies measuring DNA methylation in LINE-1 elements, no statistically significant association was observed. The two studies measuring DNA methylation in Alu elements [98,100] showed consistent statistically significant inverse associations with oxychlordane, p,p′-DDE and DDT. Increasing PCB183, heptachlor epoxide, trans-nonachlordane, and PBDE47 in a study population from Korea (*N* = 86) and PCB 156, 99, and 105, β-HCH, α-chlordane, mirex, sum of PCBs, and sum of POPs in a study population from Denmark (*N* = 70) was significantly associated with lower DNA methylation in Alu elements. Consistently, in a population of Japanese women (*N* = 399), serum POPs were inversely associated with the global DNA methylation level measured by LUMA [20]. In an elder population from Sweden (*N* = 519) [99], however, increasing total and non-ortho toxic equivalency (TEQ) levels, PCB126, and p,p′-DDE concentrations was significantly associated with increasing global DNA methylation levels also measured by LUMA (*P* < 0.05) [99]. For PFCs, in a study population from the US (*N* = 671) [101], a 12 ng/mL increase in PFOS levels was associated a difference of 20% (95% CI 0.09 to 0.32) in average 5-mC levels. Other PFCs did not show statistically significant associations. In one epigenome-wide association study in the SMART study population (*N* = 35) [29], only one CpG site in the *TSP50* gene promoter showed increased DNA methylation with elevated BPA exposure under the established significance threshold of minimum absolute change of 10% by BPA levels and a *P* value <0.05. In pyrosequencing validation, a region in this gene showed a 26% decrease in average percent methylation (*P* value = 0.005) comparing individuals above and below 2.39 μg/L of serum unconjugated BPA concentrations. In this study, no statistically significant regions were reported after controlling for a false discovery rate [29].

**Table 4:** **Studies of persistent organic pollutants (POPs) and other endocrine disruptors biomarkers and DNA methylation outcomes (6 studies available)**

**First author, year**	**Design**	**Population**	**Size**	**Men (%)** ^**a**^	**Age Range (yr)** ^**a**^	**Exposure assessment**	**Exposure categories**	**DNA methylation Assessment**	**DNA methylation endpoint**	**Chemical(s) (if PCBs, highest, lowest & median association and/or statistically significant)**	**Association**	**95% Confidence Interval or p-value**	**Adjustment Factors**
***Persistent Organic Pollutants***													
Rusiecki, 2008 [100]	CS	Greenland, Denmark (AMAP)	70	87	1967	Plasma by GC	Per log-transformed ng/g lipid increase	Peripheral leukocyte	Average % methylation		Difference		QC reported. BEE or CH assessment NR. Models adjusted for age and smoking,
						PCB 28, 52, 99, 101, 105, 118, 128, 138, 153, 156, 170, 180, 183 and 187, p,p’-DDT, p,p’-DDE, β-HCH, Hexachlorobenzene, Chlordane, Cis-chlordane, Oxychlordane, α-Chlordane, Mirex, Toxaphene, ΣPCBs, ΣPOPs		Global by quantitative pyrosequencing in:					
								LINE-1		PCB 118	–0.73	P = 0.12	
										PCB 128	–0.01	P = 0.99	
										PCB 156, 170	–0.48	P = 0.26 and 0.15	
								Alu		PCB 156	–0.66	P < 0.01	
										PCB 52	–0.12	P = 0.36	
										PCB 99, 105	–0.51	P < 0.01 both	
										p,p’-DDT	–0.26	P = 0.01	
										p,p’-DDE	–0.38	P = 0.01	
										β-HCH	–0.48	<0.01	
										Oxychlordane	–0.32	<0.01	
										α-Chlordane	–0.75	P = 0.05	
										Mirex	–0.27	P = 0.01	
										ΣPCBs	–0.56	<0.01	
										ΣPOPs	–0.48	<0.01	
Kim, 2010 [98]	CS	Uljin county, South Korea.	86	39.5	56.2 ± 7.0	Serum POPs by GC-HRMS	Per ng/g lipid increase	Whole blood.	Average % methylation		Pearson correlation		QC reported. BEE or CH assessment NR. Models adjusted for age, sex, BMI, cigarette smoking, and alcohol drinking
						PCB 74, 99, 105, 118, 138, 146, 153, 156, 157, 164, 167, 172, 177, 178, 180, 183 and 187, β-HCH, HCB, Heptachlor epoxide, Oxychlordane, trans-Nonachlor, p,p’-DDE, p,p’-DDD, p,p’-DDT, Mirex, BDE47, BDE99		Global by quantitative pyrosequencing in:					
								LINE-1		PCB 157	–0.14	p ≥ 0.05	
										PCB 146	–0.02	p ≥ 0.05	
										PCB 105, 118, 156, 172, 180	–0.07	p ≥ 0.05	
								Alu		PCB 183	–0.23	p < 0.05	
										PCB 167	–0.05	p ≥ 0.05	
										PCB 177, 178	–0.14	p ≥ 0.05	
										Heptachlor epoxide,	–0.23	<0.05	
										Oxychlordane,	–0.28	<0.05	
										trans-nonachlordane,	–0.28	<0.05	
										p,p’-DDE,	–0.29	<0.01	
										p,p’-DDT,	–0.22	<0.05	
										BDE47	–0.25	<0.05	
Lind, 2013 [99]	CS	Uppsala, Sweden (PIVUS study)	519	52	70	Serumby HRGC-HRMS	Per log-transformed ng/g lipid increase	Leukocytes	LUMA methylation index^b^		Difference		QC NR. CH assessment NR. Same age. Models adjusted for sex and smoking status..
								Global methylation by LUMA					
						PCB 74, 99, 105, 118, 126, 138, 153, 156, 157, 169, 170, 180, 189, 194, 206 and 209				Total PCB TEQ	−1.67	−3.17, −0.16	
										Non-ortho PCB TEQ	−1.76	−3.26, −0.26	
						Octachlorodibenzo-p-dioxin, HCB, TNC, p,p′-DDE, BDE47				Mono-ortho PCB TEQ	0.11	−1.37, 1.60	
										PCB 169	−3.27	−6.92, 0.37	
										PCB 206	−0.16	−3.71, 3.38	
										PCB 189	−0.56	−3.10, 1.97	
										Octachlorodibenzo-p-dioxin,	−3.19	−5.98, −0.39	
										p,p′-DDE	−2.87	−4.74, −1.00	
Itoh, 2014 [20]	CS	Japan	399	0	53.9 ±10.2	Serum by GC-HRMS	Per increase in 1 quartile categories (as an ordinal variable)	Peripheral leukocytes	1 – (LUMA methylation index^b^)				QC NR. CH assessment NR. Models adjusted for age, BMI, smoking status and alcohol drinking. Lipid-corrected values.
						PCB 17, 28, 52/69, 48/47, 74, 66, 90/101, 99, 118, 114, 105, 146, 153, 164/163, 138, 128/162, 167, 156, 182/187, 183, 177, 180, 170, 189, 202, 198/199, 196, 203, 194, 208, 206 and 209, p,p’-DDE, o,p’-DDT, p,p’-DDT, trans-Nonachlor, cis-Nonachlor, Oxychlordane, β-HCH, HCB, Mirex		Global methylation by LUMA		PCB196	−0.009	−0.38, 0.36	
										PCB74	−0.64	−1.08, −0.20	
										PCB28 and 66	−0.23	−0.59, 0.12	
										PCB17	−0.43	−0.78, −0.08	
										PCB52/69	−0.33	−0.67, −0.0007	
										PCB114	−0.46	−0.88, −0.05	
										PCB183	−0.45	−0.82, −0.07	
										p,p’-DDE,	−0.77	−1.12, −0.42	
										o,p’-DDT,	−0.75	−1.11, −0.40	
										p,p’-DDT ,	−0.83	−1.17, −0.49	
										trans-Nonachlor,	−0.44	−0.84, −0.04	
										Oxychlordane,	−0.53	−0.90, −0.15	
										β-HCH,	−0.73	−0.79, −0.35	
										HCB,	−0.41	−0.79, −0.03	
										ΣPCBs	−0.19	−0.59, 0.20	
***Perfluorinated compounds***													
Watkins, 2014 [101]	CS	Mid-Ohio River Valley, US (C8 Health Project)	671	47	41.8 (20 to 80)	Blood by HPLC separation and detection by ITMS.	Per IQR increase in mean log ng/mL levels at 2 repeated visits 5 years apart	Peripheral leukocyte			Difference		QC and CH assessment NR. Models adjusted for age, gender, BMI, smoking and current drinker status
								Global by quantitative pyrosequencing in LINE-1	Average % methylation				
						PFOA	106 ng/mL				−0.041	−0.098, 0.016	
						PFOS	12 ng/mL				0.204	0.090, 0.318	
						PFNA	0.8 ng/mL				0.064	−0.030, 0.158	
						PFHxS	2.6 ng/mL				0.020	−0.051, 0.091	
***Bisphenol A***													
Hanna, 2012 [29]	CS	U.S. (Study of Metals and Assisted Reproductive Technologies [SMART])	35	0	Mean 36 (28 to 44)	Serum	Above and below the median	Whole blood DNA					Normalization. QC reported. BEE NR. CH partially addressed. Data unadjusted. MCC NR.
						Unconjugated BPA by HPLC							
						Median =2.39 μg/L		Site specific Illumina GoldenGate and bisulfite pyrosequencing of significant regions^b^	1,505 CpG sites % methylation		A trend towards hypomethylation if difference score > |30| (p < 0.05)		
											TSP50		
											26% decrease in mean DNA m	P = 0.005	
											*r* pearson = -0.51	P = 0.001	
								Global by bisulfite pyrosequencing of LINE-1			~0.2% increase in median DNAm	P = 0.56	

CH: cell heterogeneity; BDE, polybrominated diphenyl ether; BEE: batch effect evaluation; BMI: body mass index; CDT, Comparative Toxicogenomics Database; CC: case-control; CI: confidence interval; CO: cohort; CS: cross-sectional; DNAm: DNA methylation; DDT, dichlorodiphenyl trichloroethane; DDE, dichlorodiphenyldichloroethylene; GC: gas chromatography; HPLC: high-performance liquid chromatography; HRGC-HRMS: high-resolution chromatography coupled to high-resolution mass spectometry; HRMS: high resolution mass spectrometry; IQR: interquartile range; ITMS: isotope-dilution tandem mass spectrometry; LOD: limit of detection; LUMA: Luminometric Methylation Assay; MCC: multiple comparisons correction; NR: not reported; PBDEs, polybrominated diphenyl ether; QC: quality control.

^a^Sociodemographic data available in the article, not necessarily in the subsample without missing values in DNA methylation or exposures.

^b^Significance was defined as a difference score > |13| (p < 0.05) and >10% absolute difference between the means for each group. b LUMA methylation index ranges from 1 (fully demethylated DNA) to 0 (fully methylated DNA).

All studies tested at least five POPs, but only one study [100] reported addressing multiple testing due to the elevated number of compounds. Most studies addressed potential confounding by sex, age, and smoking status [20,98,99,101]. One study did not adjust for sex, although the proportion of women was low [100]. One study presented unadjusted results [29]. POPs are highly lipophilic and their serum concentrations are closely related to serum lipid levels. Therefore, it is common practice to correct POP levels by lipid levels (that is, divide POP concentrations by total lipid concentrations). Alternatively, some authors argue that lipid correction may be problematic under certain assumptions [102]. In addition to lipid correction, it is advisable to conduct sensitivity analyses to evaluate robustness of findings using different approaches of handling lipid adjustment, such as conducting separate adjustment for total lipid levels with lipid-uncorrected POPs in regression settings. All retrieved studies evaluating POPs only conducted analyses with lipid-corrected concentrations. Both standardization of summary POP measurements (TEQ versus measured values or sum of POPs functional subgroups) and adjustment for lipid levels are ongoing challenges that require consensus in order to facilitate data comparison and meta-analysis. No study reported evaluation of the potential confounding effect of tissue cell heterogeneity.

##### Polycyclic aromated hydrocarbons and DNA methylation

PAHs are widespread environmental contaminants from incomplete combustion of organic materials such as fossil fuels, which are comprised of two or more fused benzene rings arranged in various configurations [103]. PAH metabolites in human urine, including 1-hydroxypyrene (1-OHP), 1-hydroxypyrene-O-glucuronide, 3-hydroxybenzo[a]pyrene, 7,8,9,10-tetrahydroxy-7,8,9, 10-tetrahydrobenzo[a]pyrene, and a other hydroxylated PAHs, can be used as biomarkers of internal dose to assess recent exposure to PAHs [104]. Development of biomarkers of exposure to PAHs and related compounds includes detection of protein and DNA adducts, which can be interpreted as indicators of effective dose [105]. The occurrence of PAHs in ambient air, food, drinking water, tobacco smoke, automobile exhausts, dust, and contaminated air from occupational settings [106,107] is an increasing concern for general populations given their carcinogenicity and other reported potential health effects including allergy, asthma, cardiovascular, and respiratory diseases [108]. The causative mechanisms of PAH-related health effects on the molecular level are not completely understood, and epigenetic mechanisms may be involved. Benzo[a]pyrene (BaP) has been reported to disrupt DNA methylation patterns in experimental models [109,110]. In breast cancer cell lines, BaP treatment was related to hypomethylation events at a number of repeat elements [109]. BaP induced a 12% decrease in total 5-mC content of cellular DNA of BALB/3 T3 mouse cells [110]. BaP exposure to zebrafish embryos significantly decreased global DNA methylation by 44.8% [111]. Binding of BaP adducts to DNA decreased methylation by reducing binding and activity of DNMTs [112,113]. Interestingly, experimental evidence suggests that PAH-DNA adduct formation may preferentially target methylated genomic regions [114-117] that may interfere their DNA methylation status. As a result, the interpretation of BPDE-adducts as indicators of effective dose in studies of DNA methylation is not clear.

We identified three publications investigating the association between PAHs and DNA methylation (Table 5). These studies were conducted in Mexico [118], Poland [119], and China [120]. PAH exposure was measured in urine as 1-hydroxypryene [118,120] or 1-pyrenol [119] and in peripheral blood leukocytes as anti-B[a]PDE-DNA adducts [119]. Global DNA methylation was assessed by quantitative pyrosequencing of LINE-1 and Alu elements in two studies [118,119]. CpG site-specific DNA methylation was measured in candidate genes by quantitative pyrosequencing in two studies [118,119] and by methylation-specific quantitative PCR in one study [120]. In the Polish study population (*N* = 92) [119], increasing levels of blood and urine exposure biomarkers were associated with increasing DNA methylation in LINE-1 and Alu elements (all *P* values <0.004). In contrast, in the Mexican study population (*N* = 39), urine 1-hydroxypyrene was inversely associated with LINE-1 and Alu elements [118]. The associations, however, were not statistically significant. The two studies evaluating DNA methylation in candidate regions by quantitative pyrosequencing showed consistent directions in the associations with increasing exposure biomarkers levels in genes *p53* and *IL-6* [118,119]. The associations, however, were statistically significant only in the Polish study (absolute difference in average percent 5-mC per unit increase in urine exposure biomarker was −1.58% (*P* < 0.001) in *p53* and 1.06% (*P* = 0.012) *IL-6* genes) [119]. In the Mexican population, the difference in average percent 5-mC was −1.57% (95% CI −2.9%, −0.23%) for a genomic region in *IL-12* [118]. In the Chinese study population (*N* = 128), the *p16*^*INK4α*^ promoter methylation measured by methylation-specific quantitative PCR [120] showed a positive correlation with urine 1-hydroxypyrene (Spearman *r* = 0.45, *P* < 0.001), which was not consistent with the nonsignificant results from the Polish study [119]. Only one study addressed potential confounding including sex, age, and smoking status [118]. No study reported evaluation of potential confounding effect of tissue cell heterogeneity.

**Table 5:** **Studies of PAH exposure biomarkers and DNA methylation outcomes (3 studies available)**

**First author, year**	**Design**	**Population**	**Size**	**Men (%)** ^**a**^	**Age Range (yr)** ^**a**^	**Exposure assessment**	**Exposure categories**	**DNA methylation Assessment**	**DNA methylation endpoint**	**Association**	**95% Confidence Interval or p-value**	**Data pre-processing and adjustment factors**
Alegria-Torres, 2013 [118]	CS	San Luis Potosi, Mexico. Occupational population	39	100	42.5 (16 to 75)	Urine	Per μg/g increase	Peripheral leukocytes	Average % methylation			QC or CH assessment NR. Models adjusted for smoking status, usual alcohol drinking, current medication, age, and average number of cigarettes smoked.
						1-Hydroxypyrene by HPLC		Quantitative Pyrosequencing		Difference		
						(Mean=0.18 μg/g creatinine)		Specific				
								Interleukin 12		−1.57	−2.9, −0.23	
								p53		−2.7	−5.46, 0.06	
								TNF-α		−3.9	−8.28, 0.48	
								IFN-γ		−0.43	−16.45, 15.59	
								IL-6		0.22	−9.19, 9.63	
								Global				
								LINE-1		−0.49	−4.74, 3.76	
								Alu		−0.55	−1.25, 0.16	
Pavanello, 2009 [119]	CS	Poland	92	100	37 (20-59)	Urine		Peripheral blood lymphocytes	Average % methylation	Difference		QC reported. CH assessment or adjustment for potential confounders NR. All participants were non-current smokers
						1-pyrenol by HPLC-F	Per μmol/mol creatinine increase	Quantitative pyrosequencing				
						Peripheral blood lymphocites		Specific				
								p53		−1.58	P < 0.001	
								p16		−0.01	P = 0.736	
								HIC1		−0.57	P = 0.059	
								IL-6		1.06	P = 0.012	
								Global		0.72	P = 0.01	
								LINE-1		0.13	P = 0.004	
								Alu				
						Anti-BPDE–DNA by HPLC-F analysis of BP-tetrol-I-1	Per adducts /10^8^ nucleotides increase	Specific				
								p53		−1.04	P < 0.001	
								p16		−0.02	P = 0.314	
								HIC1		−0.31	P = 0.142	
								IL-6		0.57	P = 0.043	
								Global				
								LINE-1		0.63	P < 0.001	
								Alu		0.10	P < 0.001	
Yang, 2012 [120]	CS	Anshan City, Liaoning, China	128	100	42.07	Urine	Log transformed μg/L	Peripheral blood lymphocytes	% methylation			QC or CH NR. Unadjusted for potential confounders.
						1-Hydroxypyrene		Specific by methylation specific quantitative PCR				
						(Overall mean=6.56)		p16^INK4α^		*r* spearman = 0.450	<0.001	

AAS, atomic absorption spectrometry; BaP, benzo[a]pyrene; CH: cell heterogeneity; HPLC, high-performance liquid chromatography; HPLC-F, high-performance liquid chromatography–fluorescence; IQR, interquartile range; LOD, limit of detection; NR: not reported; QC: quality control.

^a^Sociodemographic data available in the article, not necessarily in the subsample without missing values in DNA methylation or exposures.

#### General discussion and needs for future epidemiologic research

Epidemiologic evidence from distinct study populations suggests a trend for an association between increasing cadmium exposure with increased DNA methylation and a trend for an association between increasing lead and POP exposures with decreased DNA methylation, although additional studies are needed to confirm those trends. For other environmental chemicals, the low number of studies did not allow to recognize patterns in their associations with measures of DNA methylation. The epidemiologic associations were mostly in agreement with experimental evidence, although additional work is needed to better understand the relevance of the dose levels and routes of administration used in experimental studies in the context of human exposure. While the limited number of studies and the heterogeneity in DNA methylation markers limit the conclusion of this review, the evidence accrued so far supports the importance of environmental exposures in modulating the epigenome.

A limitation of the review was the substantial heterogeneity in the assessment methods of DNA methylation, especially for studies reporting global DNA methylation, which challenged the comparability across studies. For instance, LINE-1 and Alu repetitive elements have been classically used as a surrogate marker for global DNA methylation because they are abundant, hypermethylated, regions in the genome (more than one third of DNA methylation in these repetitive elements) [121]. LINE-1 and Alu elements, however, could be regulated by specific mechanisms and respond specifically to cellular stressors [122]. Other studies assessed DNA methylation globally by estimating the percentage of methylated DNA over the total number of genomic cytosines [19] or the LUMA methylation index [20,99] that goes from 0 (fully methylated DNA) to 1 (fully demethylated DNA). Among studies reporting absolutes differences in global DNA methylation, the strength of the statistically significant associations ranged between a difference (absolute value) in DNA methylation percent of 0.25 in LINE-1 per IQR (19 μg/g) of patella lead [68] to 0.75 in Alu per log ng/g lipid increase of α-Chlordane concentrations [100]. Among studies reporting differences in the relative scale, the corresponding associations ranged from a relative change of 14% comparing tertiles 3 to 1 of lead [69] to 75% comparing participants above and below the median cadmium levels [19]. Some of the retrieved studies reported dose-responses using flexible approaches (that is, quantile categories or nonparametric splines) and mostly showed fairly monotonic relationships of DNA methylation with cadmium [55], lead [68,69], and POPs [20,98-100], which add further significance to the findings.

Overall, the temporality of the reported associations cannot be evaluated in this systematic review given the low number of prospective studies. Among the four studies with originally prospective designs [19,55,68,101], all of them reported cross-sectional analyses with samples for DNA methylation and exposure status determination collected at the same time point. Only two of the prospective studies [19,101] included repeated measurements and additionally reported prospective associations of baseline exposures with DNA methylation in samples collected at follow-up visits. For cadmium, the cross-sectional association with global DNA methylation was statistically significant, whereas the prospective association after 10-year follow-up was not [19]. For PFCs, the associations with DNA methylation measured at the end of follow-up were reported not to be different either using biomarkers from samples collected at enrollment, at the end of follow-up, or the average of both [101]. The relevant type of exposure (short term versus long term), latency time, and persistence of the potential epigenetic effects of individual environmental chemicals in human populations, however, are unknown and may differ by compound. Future longitudinal studies with sufficient repeated measurements over time, which can enable the evaluation of trends and trajectories of DNA methylation by environmental exposures levels, are needed.

A major challenge in the evaluation of the association between environmental chemicals and DNA methylation was the heterogeneity of adjustment for potential confounders. For instance, residual confounding by smoking is a typical concern in epidemiologic studies assessing potential environmental chemical-epigenetic effects, because tobacco smoke is a major source of chemicals, including cadmium, lead, and PAHs, and others [32] that can have potential epigenetic effects. Most, but not all [29,53,69,85], of the retrieved articles assessed potential confounding by smoking. Sex and age are important sociodemographic factors that must be also considered as potential confounders, since they have also been related to differences in DNA methylation [123,124]. Only three studies did not address confounding by both sex and age [29,69,119]. In addition to adjustment in regression models, an alternative strategy to evaluate residual confounding is to perform separate analyses in subgroups of interest, for instance sex or smoking. For cadmium, one study in Argentinean women [54] the study population was mostly made of never smokers. For PAH, one study reported levels of DNA methylation separately for smokers and non-smokers with no statistically significant differences [120], and another study reported that all participants were not current smokers [119]. For POPs, two studies evaluated findings in smoking status subgroups [99,100], with no significant differences in the estimated associations. Four studies stratified by sex [85,99-101], reporting similar results in men and women, except a study of mercury in dental professional [85] that found a significant association between hair mercury and *SEPP1* hypomethylation only among males. Eight study populations were made only of adult men or women [20,29,53,54,68,118-120]. In addition to sex and smoking, four articles additionally performed subgroup analysis by candidate polymorphisms [29,30,54,99] mostly in genes from one-carbon metabolism and exposure-related pathways (that is, polymorphisms in the Ah receptor for POPs). In addition to candidate genes, there is mounting evidence now supporting a role of the genetic variation in cis in determining DNA methylation status [124,125]. For site-specific methylation, thus, it is advisable to evaluate whether the observed associations may be attributed to nearby polymorphisms, which may be unbalanced by exposure levels by chance. Only one study reported evaluation of SNP-related clustering of DNA methylation [53]. Another study incorporated into the analysis genotypes from SNPs known to determine DNA methylation in the significant regions of interest [29].

Artifactual variation from DNA isolation and processing and methylation assessment methods and tissue-specific nature of DNA methylation profiles are other sources of potential biases. It is well established now that differential tissue-type cell heterogeneity [123,126] and, for large studies and studies using ‘omics’ technologies for DNA methylation assessment, evaluation and correction of potential batch effects [127,128] and background correction and normalization methods [128,129] are compelling issues that must be addressed and adequately reported. Only five studies are reported addressing tissue-specific cell heterogeneity [19,30,53,68,99]. None of the two studies using microarrays technologies reported evaluating potential batch effects [29,53]. Moreover, in the specific case of microarray technologies, given the large numbers of statistical tests conducted, it is usually required to correct for multiple comparisons. The only microarray-based study reporting methods to address multiple comparisons found no significant associations after controlling of the false positive rate, something expected given the small sample size of that study (*n* = 17) [53]. A total of three [54,69,120] studies attempted to validate significant regions either by using alternative DNA methylation assays to assess the consistency of results and/or by conducting functional assays in experimental models. While there is evidence supporting that arsenic-related methylation changes are associated with changes in gene expression [12,130], for other environmental chemicals, the available epidemiologic evidence is limited. Only one of the reviewed studies for cadmium [54] had available genome-wide gene expression measurements. However, the association of changes in DNA methylation with gene expression was not directly evaluated for the cadmium-related epigenetic regions. Epidemiologic studies that include assessments of environmental chemicals and coupled DNA methylation and gene expression data are needed.

An emerging issue relates to the inability of sodium bisulfite conversion, which is the commonly used method for determination of 5-mC at single-base resolution, to distinguish 5-mC from its oxidative derivative 5-hydroxymethylcytosine (5-hmC) [131]. It has been reported that 5-hmC is enriched in intergenic regions, including LINE-1 elements and gene body regions [132-134]. In studies evaluating the association of DNA methylation and environmental chemicals using bisulfite conversion based methods for DNA methylation assessment, residual measurement error by 5-hmC content is, thus, possible. In one of the reviewed papers [19], which measured global DNA methylation and hydroxymethylation in human blood samples (using antibodies specific for 5-mC and 5-hmC with no cross-specificity), there was a positive and statistically significant correlation between both epigenetic marks. Moreover, the direction of the association of both epigenetic markers with diverse determinants, including some metals, was mostly consistent. In addition, there is increasing evidence that 5-hmC could also play a role in epigenetic regulation of gene expression and be associated with disease susceptibility [135,136]. The health implications of the relationship between DNA methylation and hydroxymethylation in differentiated tissues are currently unknown. Advanced technology for high-throughput parallel sequencing on 5-mC and 5-hmC profiling across the genome may help to understand the role of DNA hydroxymethylation and its determinants in health.

Finally, the role of pre-natal exposure to environmental chemicals as a determinant of DNA methylation was out of the scope of this systematic review. Given the relevance of potential heritability of DNA methylation changes and post-birth effects of maternal environmental exposures, we briefly summarize here the epidemiologic evidence that reported results on the association of maternal exposure biomarkers and DNA methylation in cord blood and was excluded as a result of secondary exclusion criteria. We identified two studies focusing on cadmium [26,53] and POPs [22,24], respectively, and one study investigating lead [25] and PAHs [23], respectively. These studies overall support an association of pre-natal exposure to environmental chemicals with epigenetic markers in the offspring, but specific systematic reviews are needed.

## Conclusions

Increasing evidence supports the role of environmental chemicals in DNA methylation changes. For cadmium, lead, and POPs, the evidence could be classified as ‘suggestive but insufficient’ considering some consistency and evidence of a dose-response relationship across studies, biological plausibility from experimental findings, and adjustment of confounding in epidemiologic studies. However, we finally concluded that for all the environmental chemicals evaluated, including cadmium, lead, and POPs, the current evidence is ‘insufficient’ to support causality given the heterogeneity among epidemiologic studies in potential for residual confounding of the associations, differences in DNA methylation assessment methods and, random error, especially because of the limited sample sizes. Important questions include the need for larger and longitudinal studies with repeated measures, validation and replication of findings, the relevance of epigenetic markers recently gaining attention such as DNA hydroxymethylation, the systematic evaluation of the dose-response relationships, and the investigation of the role of genetic variation. An emerging area of research is the role of joint exposures in changing DNA methylation, although statistical methods to comprehensively tackle mixtures of compounds are needed. As large cohorts with available measurements of environmental chemicals and genome-wide DNA methylation data become increasingly available, collaborative meta-analyses will enable to disentangle the role of environmental chemicals as determinants of DNA methylation and, also, to test the hypothesis that genomic DNA methylation may mediate chemical-related health effects.

## Additional files

Additional file 1: Table S1. PubMed search strategies for environmental chemicals and DNA methylation. Additional file 2: Table S2. Study quality criteria (17 studies included in the current review).

## Abbreviations

1-OHP: 1-hydroxypyrene
5-hmC: 5-hydroxymethylcytosine
5-mC: 5-methylcytosine
AAS: atomic absorption spectrometry
BaP: benzo[a]pyrene
BDE: polybrominated diphenyl ether
BEE: batch effect evaluation
BMI: body mass index
BPA: bisphenol A
CC: case-control
CDT: Comparative Toxicogenomics Database
CH: cell heterogeneity
CI: confidence interval
CO: cohort
CS: cross-sectional
DDE: dichlorodiphenyldichloroethylene
DDT: dichlorodiphenyl trichloroethane
DNAm: DNA methylation
GC: gas chromatography
HPLC: high-performance liquid chromatography
HPLC-F: high-performance liquid chromatography-fluorescence
HRGC-HRMS: high-resolution chromatography coupled to high-resolution mass spectrometry
HRMS: high-resolution mass spectrometry
IQR: interquartile range
LUMA: Luminometric Methylation Assay
MCC: multiple comparison correction
NR: not reported
PAH: polycyclic aromatic hydrocarbons
PBDEs: polybrominated diphenyl ether
PCBs: polychlorinated biphenyls
PFC: perfluorinated compounds
PFOA: perfluorooctanoic acid
PFOS: perfluorooctanesulfonic acid
POPs: persistent organic pollutants
TEQ: toxic equivalency
