# Supplementary material for: Environmental chemicals and DNA methylation in adults: a systematic review of the epidemiologic evidence
Source: Clin Epigenetics. 2015 Apr 29;7(1):55. doi: 10.1186/s13148-015-0055-7 (PMC4433069; doi:10.1186/s13148-015-0055-7)
Supplement: Additional file 1: Table S1. — PubMed search strategies for environmental chemicals and DNA methylation. [file 13148_2015_55_MOESM1_ESM.pdf]

Table S1. PubMed search strategies for environmental chemicals and DNA methylation

Database	PubMed
Date	April 10, 2014
Strategy	We combined the results for each environmental chemical search strategy (#1 to #10 below) with the results for the DNA methylation search (#11 below)
#1. Cadmium [73]	"cadmium"[Mesh] OR “cadmium”
#2. Lead [26]	"Lead Poisoning"[Mesh] OR "Lead"[Mesh]
#3. Nickel [68]	“Nickel”[Mesh] OR “Nickel”
#4. Mercury [29]	“Mercury”[Mesh] OR “Mercury”
#5. Tungsten [1]	“Tungsten”[Mesh] OR “Tungsten”
#6. Antimony [1]	“Antimony”[Mesh] OR “Antimony”
#7. Persistent organic pollutants (POPs) [156]	("Polychlorinated Biphenyls"[Mesh] OR "Hydrocarbons, Chlorinated"[Mesh] OR "Dioxins"[Mesh] OR "Halogenated Diphenyl Ethers"[Mesh] OR "Polybrominated Biphenyls"[Mesh] OR "perfluorooctane sulfonic acid"[Substance Name] OR "perfluorooctanoic acid"[Substance Name] OR "Carbon Tetrachloride"[Mesh] "Polychlorinated Biphenyls" OR "chlorinated hydrocarbons" OR aldrin OR "carbon tetrachloride" OR chlordane OR chlordecone OR chlorobenzene* OR hexachlorobenzene OR chloroform OR ddt OR dichlorodiphenyltrichloroethane OR dichloroacetate OR "dichlorodiphenyl dichloroethylene" OR dichlorodiphenyldichloroethane OR dichloroethylenes OR dieldrin OR endrin OR "ethyl chloride" OR "ethylene dichlorides" OR heptachlor OR lindane OR hexachlorocyclohexane OR methoxychlor OR "methyl chloride" OR "methylene chloride" OR mirex OR mitotane OR "picryl chloride" OR polychloroterphenyl OR tetrachloroethylene OR toxaphene OR trichloroepoxypropane OR trichloroethane* OR trichloroethylene OR "vinyl chloride" OR "Dioxins" OR TCDD OR "Halogenated Diphenyl Ethers" OR "diphenyl ethers" OR PBDE* OR PCDE* OR "Polybrominated Biphenyls" OR "polybrominated biphenyls" OR Polybromobiphenyl* OR "polychlorinated biphenyls" OR Polychlorobiphenyl OR PCB OR "perfluorooctane sulfonic acid" OR "perfluorooctane sulfonic acid" OR pfosa OR 1763-23-1 OR "perfluorooctane sulfonate" OR "perfluorooctanoic acid" OR 335-67-1 OR "perfluorooctanoic acid" OR PFOA OR "pentadecafluorooctanoic acid" OR "perfluorooctanoyl chloride" OR "sodium perfluorooctanoate" OR "perfluorinated octanoic acid" OR "Carbon Tetrachloride")
#8. Phthalates [66]	"phthalic acid"[Substance Name] OR "Phthalic Acids"[Mesh] OR phthalate* OR "phthalic acid" OR phthalate* OR "phthalic acids" OR "dibutyl phthalate" OR "diethylhexyl phthalate" OR "o-phthalaldehyde" OR "phthalic anhydrides" OR phthalimides OR thalidomide
#9. Bisphenol A [64]	"bisphenol A"[Substance Name] OR "bisphenol A"
#10. Polycyclic aromated hydrocarbons (PAHs) [383]	"Polycyclic Hydrocarbons, Aromatic"[Mesh]
#11. DNA methylation	"DNA methylation" [Mesh] OR "epigenomics" [Mesh] OR ((“DNA” [Mesh] OR “Deoxyribonucleic acid“) AND (“methylation” [Mesh] OR “methylation”)) OR “DNA methylation” OR “epigenomics” OR “epigenetics”

The number in brackets after each chemical is the number of articles identified based on a chemical-specific search strategy after combining the search strategy for this chemical with the search strategy for DNA methylation (#11).
