# Supplementary material for: Environmental chemicals and DNA methylation in adults: a systematic review of the epidemiologic evidence
Source: Clin Epigenetics. 2015 Apr 29;7(1):55. doi: 10.1186/s13148-015-0055-7 (PMC4433069; doi:10.1186/s13148-015-0055-7)
Supplement: Additional file 2: Table S2. — Study quality criteria (17 studies included in the current review). [file 13148_2015_55_MOESM2_ESM.pdf]

Table S2. Study Quality Criteria (17 studies included in the current review)

	Rusiecki et al. (2008) [1]	Pavanello et al (2009) [2]	Wright et al. (2010) [3]	Kim et al (2010) [4]	Yang et al. (2012) [5]	Hossain et al. (2012) [6]	Hanna et al. (2012) [7]	Li et al (2013) [8]	Goodrich et al. (2013) [9]	Zhang et al. (2013) [10]	Lind et al. (2013) [11]	Tajuddin et al. (2013) [12]	Alegria-Torres et al. (2013) [13]	Sanders et al. (2014) [14]	Tellez-Plaza et al. (2014) [15]	Watkins et al. 2014 [16]	Itoh et al. 2014 [17]
All studies																	
Was exposure assessed at the individual level?	■	■	■	■	■	■	■	■	■	■	■	■	■	■	■	■	■
Was exposure assessed using a biomarker?	■	■	■	■	■	■	■	■	■	■	■	■	■	■	■	■	■
Were QC data (reliability or validity and LOD) in biomarker data provided?	▒	■	■	■	□	■	▒	□	▒	□	▒	□	■	▒	■	□	■
Did the authors provide any information on QC control for DNA methylation assessment methods?	■	■	■	■	□	■	■	■	■	■	□	■	□	▒	■	□	■
Was the intensity of search of DNA methylation changes independent of exposure status?	■	□	■	■	□	■	■	□	■	□	■	■	■	■	■	■	■
Were the data collected in a similar manner for all participants?	■	□	■	■	■	■	■	□	■	■	■	■	■	■	■	■	■
Were the same exclusion criteria applied to all participants?	□	□	□	■	■	■	□	□	■	■	■	■	■	■	■	■	■
Was the time period over which collected samples for DNA methylation and exposure status determination the same?	■	□	■*	■	■	■	■	■	■	■*	■	■	■	■	■**	■**	■
Was the interviewer blinded with respect to the DNA methylation or exposure status of the person interviewed?	■	□	■	■	□	■	■	□	■	□	■	■	■	■	■	■	■
Did the authors present internal comparisons within study participants?	■	■	■	■	■	■	■	■	■	■	■	■	■	■	■	■	■
Did the authors address potential confounding factors for DNA methylation in addition to age, sex and cell heterogeneity?	▒	□	■	▒	□	▒	▒	□	▒	▒	▒	■	▒	■	■	▒	■
Studies with micro-array DNA methylation data. Did the authors provide any information on the following items?																	
a. Criteria for samples allocation in the array	-	-	-	-	-	-	□	-	-	-	-	-	-	□	-	-	-
b. Criteria for a priori probes exclusion (i.e. if based on low quality scores, presence of SNPs)	-	-	-	-	-	-	■	-	-	-	-	-	-	▒	-	-	-

Environmental Chemicals and DNA methylation: a Systematic Review of Epidemiologic Evidence
Adrian Ruiz, Chin-Chi Kuo, Pilar Rentero, Wan-Yee Tang, Josep Redon, Jose M. Ordovas, Ana Navas-Acien, and Maria Tellez-Plaza *
* Institute for Biomedical Research Hospital Clinic of Valencia - INCLIVA (Spain) and Department of Environmental Health Sciences, Johns Hopkins Bloomberg School of Public Health (Baltimore, US); maria.tellez@uv.es; mtellez3@jhu.edu

c. Batch effect evaluation and/or correction	-	-	-	-	-	-	□	-	-	-	-	-	-	□	-	-	-
d. Normalization method	-	-	-	-	-	-	■	-	-	-	-	-	-	■	-	-	-
e. Visualization of potential SNP clustering in statistically significant regions	-	-	-	-	-	-	▪	-	-	-	-	-	-	□	-	-	-
f. Validation (replication or sequencing) of significant regions	-	-	-	-	-	-	■	-	-	-	-	-	-	■	-	-	-

■ fulfilled; ▪ partially fulfilled; □ not fulfilled or information not available; - not applicable. * Prospective design although reported associations are cross-sectional. ** Both cross-sectional and prospective associations reported.

REFERENCES

1. Rusiecki JA, Baccarelli A, Bollati V, Tarantini L, Moore LE, Bonefeld-Jorgensen EC: **Global DNA hypomethylation is associated with high serum-persistent organic pollutants in Greenlandic Inuit.** *Environ Health Perspect* 2008, **116:**1547-1552.
2. Pavanello S, Bollati V, Pesatori AC, Kapka L, Bolognesi C, Bertazzi PA, Baccarelli A: **Global and gene-specific promoter methylation changes are related to anti-B[a]PDE-DNA adduct levels and influence micronuclei levels in polycyclic aromatic hydrocarbon-exposed individuals.** *Int J Cancer* 2009, **125:**1692-1697.
3. Wright RO, Schwartz J, Wright RJ, Bollati V, Tarantini L, Park SK, Hu H, Sparrow D, Vokonas P, Baccarelli A: **Biomarkers of lead exposure and DNA methylation within retrotransposons.** *Environ Health Perspect* 2010, **118:**790-795.
4. Kim KY, Kim DS, Lee SK, Lee IK, Kang JH, Chang YS, Jacobs DR, Steffes M, Lee DH: **Association of low-dose exposure to persistent organic pollutants with global DNA hypomethylation in healthy Koreans.** *Environ Health Perspect* 2010, **118:**370-374.
5. Yang P, Ma J, Zhang B, Duan H, He Z, Zeng J, Zeng X, Li D, Wang Q, Xiao Y, et al: **CpG site-specific hypermethylation of p16INK4alpha in peripheral blood lymphocytes of PAH-exposed workers.** *Cancer Epidemiol Biomarkers Prev* 2012, **21:**182-190.
6. Hossain MB, Vahter M, Concha G, Broberg K: **Low-level environmental cadmium exposure is associated with DNA hypomethylation in Argentinean women.** *Environ Health Perspect* 2012, **120:**879-884.
7. Hanna CW, Bloom MS, Robinson WP, Kim D, Parsons PJ, vom Saal FS, Taylor JA, Steuerwald AJ, Fujimoto VY: **DNA methylation changes in whole blood is associated with exposure to the environmental contaminants, mercury, lead, cadmium and bisphenol A, in women undergoing ovarian stimulation for IVF.** *Hum Reprod* 2012, **27:**1401-1410.
8. Li C, Yang X, Xu M, Zhang J, Sun N: **Epigenetic marker (LINE-1 promoter) methylation level was associated with occupational lead exposure.** *Clin Toxicol (Phila)* 2013, **51:**225-229.
9. Goodrich JM, Basu N, Franzblau A, Dolinoy DC: **Mercury biomarkers and DNA methylation among Michigan dental professionals.** *Environ Mol Mutagen* 2013, **54:**195-203.

Adrian Ruiz, Chin-Chi Kuo, Pilar Rentero, Wan-Yee Tang, Josep Redon, Jose M. Ordovas, Ana Navas-Acien, and Maria Tellez-Plaza *
* Institute for Biomedical Research Hospital Clinic of Valencia - INCLIVA (Spain) and Department of Environmental Health Sciences, Johns Hopkins Bloomberg School of Public Health (Baltimore, US); maria.tellez@uv.es; mtellez3@jhu.edu

10. Zhang C, Liang Y, Lei L, Zhu G, Chen X, Jin T, Wu Q: **Hypermethylations of RASAL1 and KLOTHO is associated with renal dysfunction in a Chinese population environmentally exposed to cadmium.** *Toxicol Appl Pharmacol* 2013, **271:**78-85.
11. Lind L, Penell J, Luttropp K, Nordfors L, Syvanen AC, Axelsson T, Salihovic S, van Bavel B, Fall T, Ingelsson E, Lind PM: **Global DNA hypermethylation is associated with high serum levels of persistent organic pollutants in an elderly population.** *Environ Int* 2013, **59:**456-461.
12. Tajuddin SM, Amaral AF, Fernandez AF, Rodriguez-Rodero S, Rodriguez RM, Moore LE, Tardon A, Carrato A, Garcia-Closas M, Silverman DT, et al: **Genetic and non-genetic predictors of LINE-1 methylation in leukocyte DNA.** *Environ Health Perspect* 2013, **121:**650-656.
13. Alegria-Torres JA, Barretta F, Batres-Esquivel LE, Carrizales-Yanez L, Perez-Maldonado IN, Baccarelli A, Bertazzi PA: **Epigenetic markers of exposure to polycyclic aromatic hydrocarbons in Mexican brickmakers: a pilot study.** *Chemosphere* 2013, **91:**475-480.
14. Sanders AP, Smeester L, Rojas D, Debussycher T, Wu MC, Wright FA, Zhou YH, Laine JE, Rager JE, Swamy GK, et al: **Cadmium exposure and the epigenome: Exposure-associated patterns of DNA methylation in leukocytes from mother-baby pairs.** *Epigenetics* 2014, **9:**212-221.
15. Tellez-Plaza M, Tang WY, Shang Y, Umans JG, Francesconi KA, Goessler W, Ledesma M, Leon M, Laclaustra M, Pollak J, et al: **Association of Global DNA Methylation and Global DNA Hydroxymethylation with Metals and other Exposures in Human Blood DNA Samples.** *Environ Health Perspect* 2014.
16. Watkins DJ, Wellenius GA, Butler RA, Bartell SM, Fletcher T, Kelsey KT: **Associations between serum perfluoroalkyl acids and LINE-1 DNA methylation.** *Environ Int* 2014, **63:**71-76.
17. Itoh H, Iwasaki M, Kasuga Y, Yokoyama S, Onuma H, Nishimura H, Kusama R, Yoshida T, Yokoyama K, Tsugane S: **Association between serum organochlorines and global methylation level of leukocyte DNA among Japanese women: a cross-sectional study.** *Sci Total Environ* 2014, **490:**603-609.

Adrian Ruiz, Chin-Chi Kuo, Pilar Rentero, Wan-Yee Tang, Josep Redon, Jose M. Ordovas, Ana Navas-Acien, and Maria Tellez-Plaza *
* Institute for Biomedical Research Hospital Clinic of Valencia - INCLIVA (Spain) and Department of Environmental Health Sciences, Johns Hopkins Bloomberg School of Public Health (Baltimore, US); maria.tellez@uv.es; mtellez3@jhu.edu
